# Predicting individual tree diameter at breast height for genetically diverse *Catalpa bungei* using nonlinear mixed-effects models and UAV LiDAR data

**DOI:** 10.3389/fpls.2025.1716546

**Published:** 2025-12-19

**Authors:** Yang Zhang, Miaomiao Zhang, Qiao Chen, Liyong Fu, Wenjun Ma, Guangshuang Duan, Xinru Fu, Ziyan Zheng, Chuangye Wu, Qingqing Wang, Yuheng Shun, Pan Li

**Affiliations:** 1 Institute of Forest Resource Information, Chinese Academy of Forestry, Beijing, China; 2 Key Laboratory of Forest Management and Growth Modelling, National Forestry and Grassland Administration, Beijing, China; 3 State Key Laboratory of Tree Genetics and Breeding , Key Laboratory of Tree Breeding and Cultivation of State Forestry Administration, Research Institute of Forestry, Chinese Academy of Forestry, Beijing, China; 4 Xinyang Normal University, Xinyang, Henan, China; 5 Wen County Institute of Forestry Science, Jiaozuo, Henan, China

**Keywords:** UAV lidar, DBH prediction, nonlinear mixed-effects model (NLME), genotype differences, *Catalpa bungei*

## Abstract

**Introduction:**

Diameter at breast height (DBH) is a key parameter for assessing tree growth, carbon storage, and ecological functions. Traditional ground surveys are inefficient, labor-intensive, and terrain-limited, making them unsuitable for large-scale monitoring. Airborne LiDAR, as an advanced remote sensing tool, provides an efficient and non-destructive method for DBH estimation. However, most existing LiDAR-based models overlook the influence of genotype differences, limiting prediction accuracy.

**Methods:**

In this study, we used data from 2,899 Catalpa bungei trees of different genotypes to develop a nonlinear mixed-effects (NLME) model that incorporates genotype as a random effect. This approach improved model generalizability by using LiDAR-derived tree height (LH) and LiDAR-derived crown diameter (LCD) as core predictors. Multiple sampling strategies were also evaluated to assess their impact on model performance.

**Results:**

The results showed that, considering genotype effects, the proposed NLME model outperformed both traditional regression models and dummy-variable models (R^2^ = 0.8624, RMSE = 1.1330, TRE = 3.9555), demonstrating the important role of genotype differences in improving model accuracy. Random sampling further improved prediction accuracy while effectively reducing measurement costs.

**Discussion:**

This research introduces a new framework for integrating genotype variability into DBH prediction models and offers valuable insights for future LiDAR-based studies in genetically heterogeneous plantations. The findings provide technical support for forest management and ecosystem monitoring, as well as a methodological foundation for predicting tree growth under varying site and genetic conditions.

## 1 Research background

*Catalpa bungei* is a high-value, native tree species in China, known for its timber, medicinal, and ornamental uses. It is primarily distributed in the middle and lower reaches of the Yellow River and the North and Central China regions, with recent introductions in southern and southwestern areas (Shi et al., 2025; Wang et al., 2016). The species exhibits rich genetic diversity, with germplasm resources spanning 19 provinces. Phenotypic and molecular marker studies have revealed significant intraspecific variation, providing a basis for the selection of superior clones (Xiao et al., 2019). Under the influence of climate warming, the potential suitable distribution zone of *Catalpa bungei* is shifting northward, indicating promising ecological restoration and afforestation value in northern and northeastern China (Shi et al., 2025).

Diameter at Breast Height(DBH) is one of the most fundamental attributes in forest ecology, providing a basis for estimating tree biomass, growth rates, and competitive interactions. It also serves as a proxy for ecological indicators such as forest productivity, structural complexity, and habitat quality. Therefore, improving DBH prediction accuracy is not only relevant to forestry practice but also directly contributes to more reliable ecological monitoring and indicator development.

Given the ecological significance of DBH, a thorough understanding of the intrinsic mechanisms driving its variation is essential for accurate modeling and ecological interpretation. The coordinated variation among crown diameter(CD), tree height(H), and DBH reflects the integrated physiological and biomechanical processes underlying tree growth. Crown expansion regulates light interception and photosynthetic capacity, thereby determining the amount of carbon assimilates available for cambial activity and secondary xylem development. A larger canopy enhances gross primary production (GPP) and promotes radial growth through greater carbon allocation to the stem (Li et al., 2014). As trees grow taller, the hydraulic path between roots and leaves lengthens, increasing water transport resistance and reducing leaf water potential; to maintain hydraulic efficiency, trees must increase sapwood area and conduit density, resulting in stem thickening (Ryan and Yoder, 1997; Mencuccini, 2003). Meanwhile, increasing height amplifies mechanical stress caused by wind and self-weight, requiring proportional increases in stem diameter to preserve structural stability and prevent bending or buckling (King, 2005). Collectively, these physiological, hydraulic, and mechanical constraints give rise to the allometric scaling relationships among H, CD, and DBH, representing an adaptive balance between carbon assimilation, water transport, and structural support in woody plants.

DBH is a critical indicator for assessing tree growth, carbon storage, and resource structure (Zhang et al., 2022; Wang et al., 2024; Ouyang et al., 2024; He et al., 2025), but traditional field measurements are time-consuming, labor-intensive, and severely constrained by topography. These limitations are especially problematic in large-scale forest monitoring. In recent years, the rapid development of remote sensing, particularly LiDAR technology, has offered an efficient and systematic solution for extracting forest structural information (Borsah et al., 2023; Balestra et al., 2024; De Conto et al., 2024), becoming a vital tool for large-scale DBH estimation. Airborne LiDAR can penetrate the forest canopy to accurately acquire three-dimensional structural parameters such as H and CD (Liu et al., 2020; Zhou et al., 2022). Compared with traditional methods, it is non-destructive, highly efficient, and suitable for complex terrains and dense forests (Dalponte et al., 2018; Oehmcke et al., 2022; Xiang et al., 2024).

Despite its advantages in structural parameter extraction, current studies using airborne LiDAR still face several challenges. Most DBH prediction models rely on fixed-effect assumptions and often overlook the potential impact of genotype variation and plot-specific conditions on tree growth. In stands with pronounced genetic heterogeneity, traditional models fail to capture the regulatory role of genotype in growth dynamics, limiting DBH estimation accuracy. Therefore, developing modeling approaches that integrate both fixed and random effects has become a critical pathway for improving DBH prediction accuracy.

In recent years, NLME models have been increasingly applied in forestry to describe the hierarchical structure of forest data and to improve prediction accuracy by accounting for both fixed and random sources of variation. They have been successfully used to model diverse forest attributes, including height–diameter relationships (Bronisz and Mehtätalo, 2020; Xu et al., 2024; Ismail et al., 2025), crown width and canopy structure (Raptis et al., 2018; Zhou et al., 2023), as well as stand biomass and growth dynamics (Qin et al., 2025; Zhu et al., 2025). These studies collectively demonstrate that incorporating stand-level or environmental variables—such as mean DBH, stand density, basal area, and climatic factors—as random effects or covariates can substantially enhance model flexibility and predictive power across forest types. The widespread adoption of NLME frameworks highlights their robustness and versatility for representing structural heterogeneity and complex ecological processes in both even- and uneven-aged forests.

Research on growth trait variation among *Catalpa bungei* genotypes has also progressed in recent years. A study systematically evaluated the growth performance of *Catalpa bungei* clones through multi-site trials. The results revealed significant genetic differences in DBH, H, and stem volume among genotypes, along with relatively high repeatability and moderate-to-high levels of genetic control. These findings provide a solid foundation for the selection and deployment of superior genotypes (Xiao et al., 2025). Additionally, Clonal trials of *Paulownia* species were conducted in both temperate and subtropical regions. The study found significant genotype-by-environment interactions in growth traits such as DBH and height, with genetic gains ranging from 18.05% to 46.03%, further confirming the critical influence of genotype on tree growth (Zhao et al., 2022).

Although NLME models have been widely applied in forestry modeling, most existing studies have treated plot, site, or stand characteristics as random effects. However, few studies have attempted to incorporate *genotypic variation* into the model structure to characterize individual growth variability and stochastic fluctuations from a genetic perspective. Previous DBH prediction models often overlooked the regulatory influence of genetic background on growth dynamics, resulting in limited prediction accuracy in stands with high genetic heterogeneity.

In this study, *genotype* was introduced as a random effect within the NLME framework for DBH modeling, enabling a systematic characterization of growth variation and random fluctuations among different genotypes. This approach substantially enhances the explanatory power and generalization ability of the model. The proposed innovation not only overcomes the limitations of conventional fixed-effect modeling but also provides a novel theoretical and technical framework for fine-scale DBH estimation and large-scale ecological monitoring in genetically diverse forest stands.

## 2 Materials and methods

### 2.1 Study area

**Figure 1:**
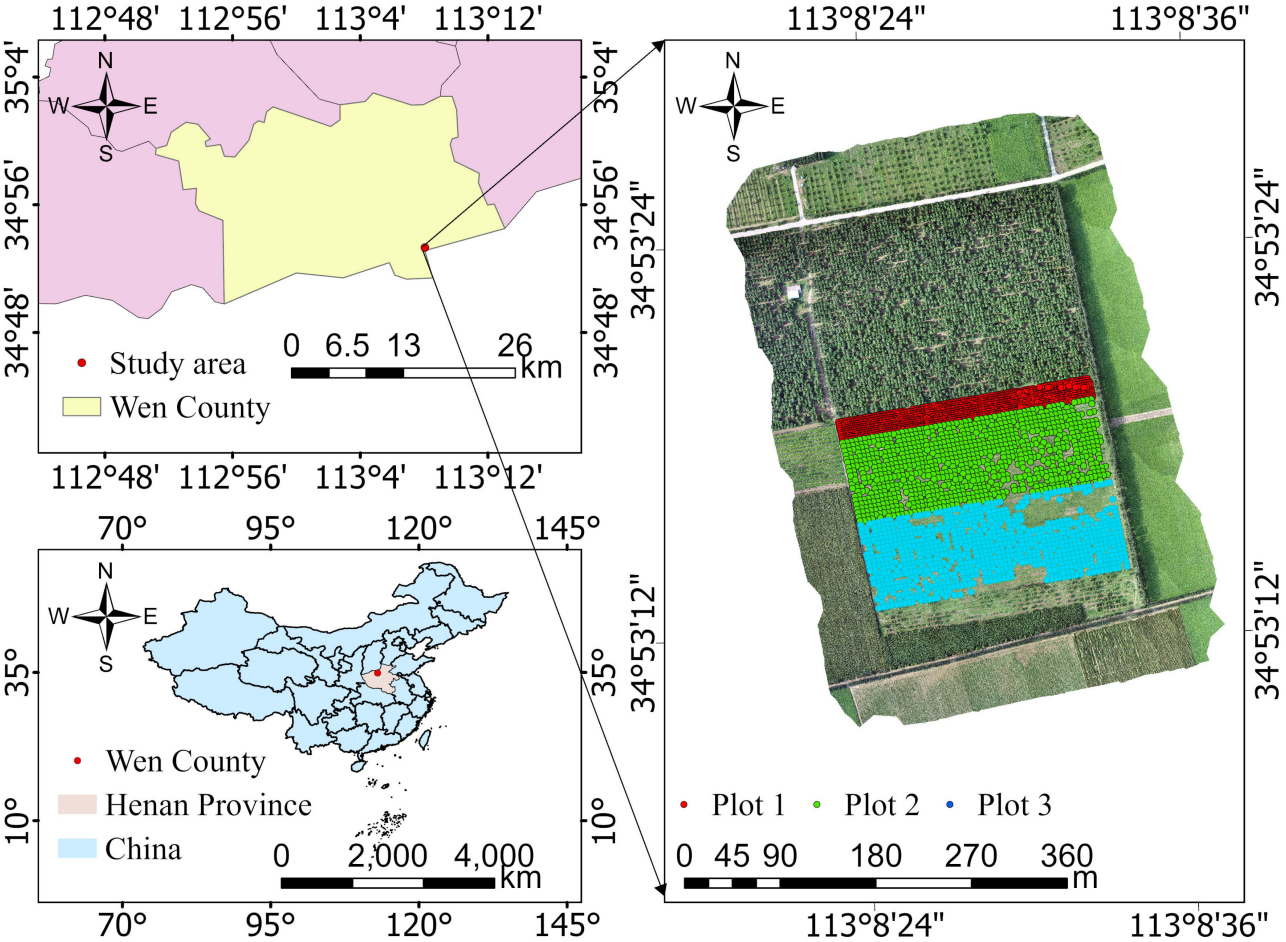
Overview of the study area.

The study area is located in Wenxian, Jiaozuo City, Henan Province, China (longitude 112°51’39”E–113°13’20”E, latitude 34°52’N–35°2’48”N), situated in the southwestern part of the North China Plain. The study area’s location and topographic features are illustrated in the map (**Figure 1**).The region lies at a relatively low elevation, ranging from 50 to 150 meters, with a terrain characterized by plains and low hills, gently sloping from northwest to southeast, forming a distinctive microclimate. The area has a temperate monsoon climate, with an average annual temperature of approximately 14 °C to 15 °C and annual precipitation between 600 and 700 mm. The predominant soil type is yellow-brown soil, which has a moderate texture and good drainage, suitable for the growth of various plant species. Wenxian, located in northwestern Henan Province and near the Yellow River, offers favorable natural conditions for ecological research and sustainable development.

**Figure 2:**
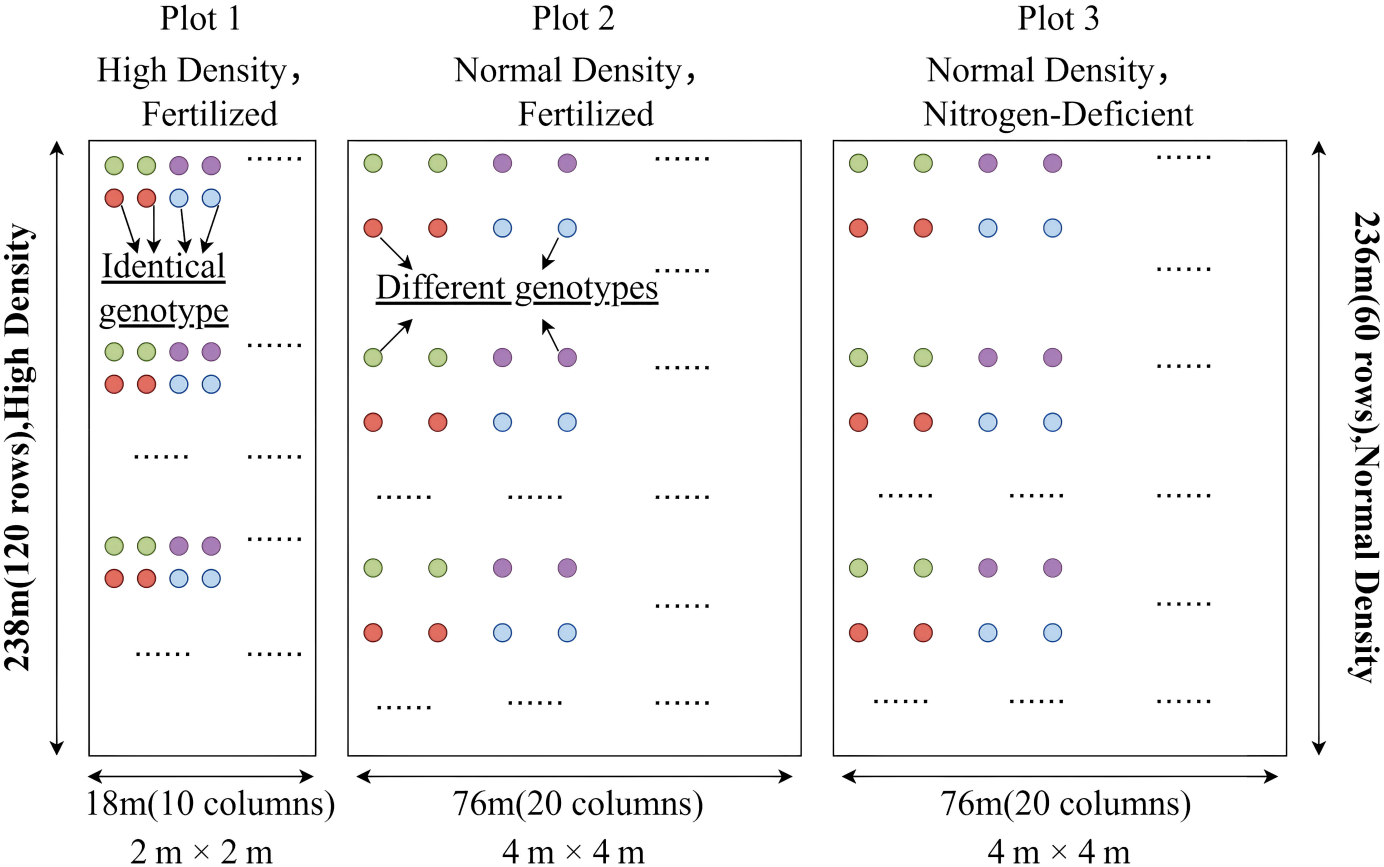
Schematic diagram of the *Catalpa bungei* treatment plots.

To investigate the growth characteristics of *Catalpa bungei* genotypes, three different treatment plots were established in the study area (**Figure 2**): Plot 1: High-density planting with standard fertilization, spacing 2 m × 2 m, arranged in 120 rows × 10 columns. A total of 200 genotypes were included, with two trees per genotype, planted in triplicate. Plot 2: Control group with standard fertilization, spacing 4 m × 4 m, arranged in 60 rows × 20 columns. A total of 200 genotypes, two trees per genotype, planted in triplicate. Plot 3: Nitrogen-deficient treatment with spacing 4 m × 4 m, 60 rows × 20 columns, also with 200 genotypes, two trees per genotype, and three replications.

### 2.2 Data collection and preprocessing

**Figure 3:**
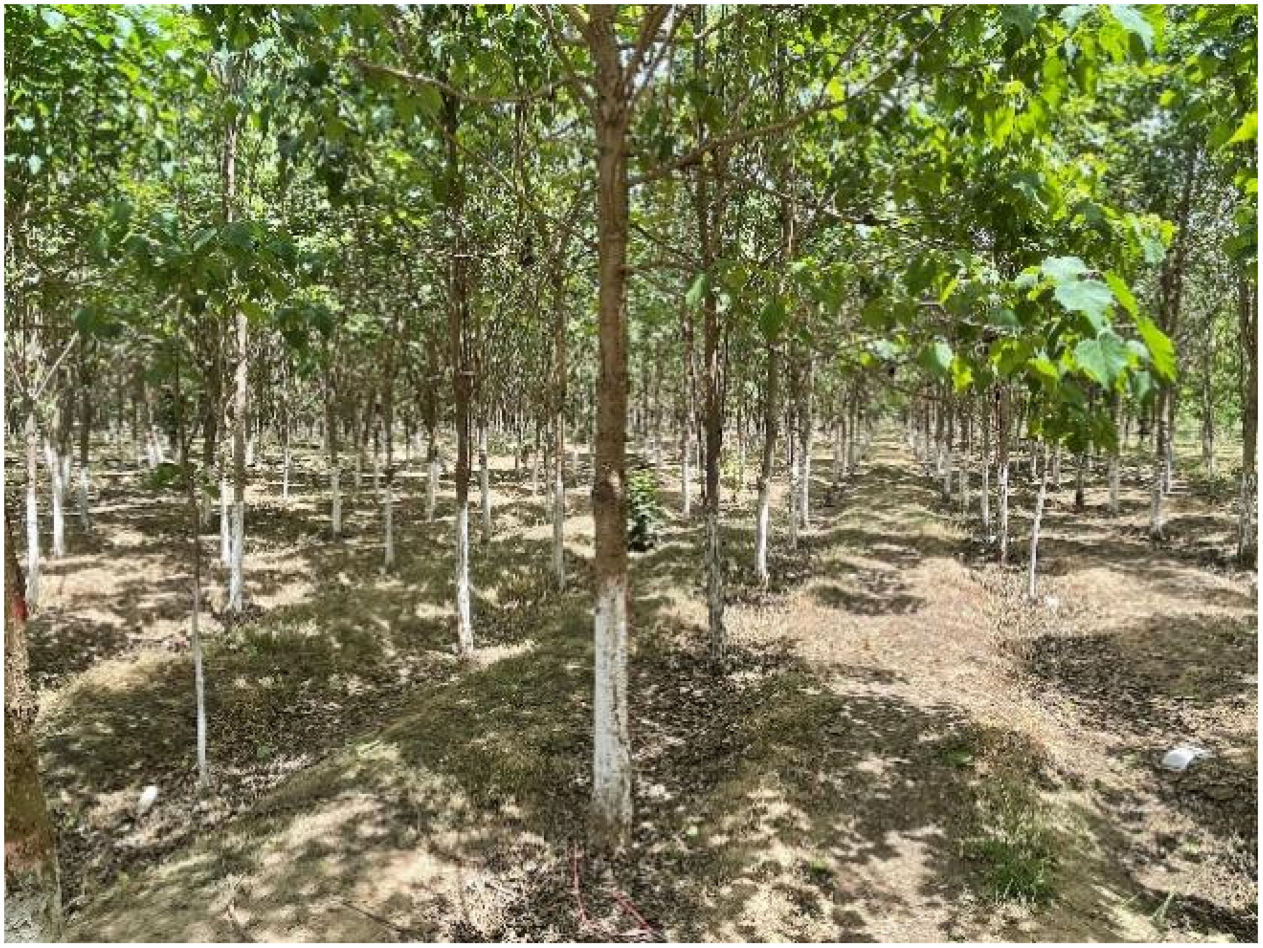
Schematic diagram of the *Catalpa bungei* treatment plots.

Ground-based data were collected during 2021, 2023, and 2024. The measurement instruments used for DBH, H, and CD include DBH calipers, telescopic height poles, and laser hypsometers. The specifications, precision, and resolution of the instruments are shown in **Table 1**. Within each plot, all upright and living Catalpa trees were measured for: DBH,H and CD. Representative vegetation types in the sample plots are shown in the field photographs (**Figure 3**). **Table 2** presents the descriptive statistics of the measured tree variables.

**Table 1:** Specifications and accuracy of ground-based measurement instruments.

Measurement indicator	Main equipment	Scale division	Accuracy
DBH (cm)	Diameter Tape	0.01 cm	± 0.2 cm
H (m)	15 m Telescopic Measuring Pole	0.1 m	± 0.5 m
CD (m)	Telescopic Crown Width Measuring Rod	0.01 m	± 0.1 m

**Table 2:** Descriptive statistics of tree variables (SD, standard deviation; DBH, diameter at breast height; H, measured tree height; CD, measured crown width; LH, LiDAR-derived tree height; LCD, LiDAR-derived crown diameter).

Variable	Mean	SD	Max	Min
DBH (cm)	4.86	3.06	14.35	0.50
H (m)	3.69	1.55	8.20	1.30
CD (m)	1.67	0.97	6.35	0.20
LH (m)	3.97	1.78	10.60	1.30
LCD (m)	1.88	1.09	6.00	0.05

UAV LiDAR data were also collected in 2021, 2023, and 2024 using the BB-4 Bumblebee UAV platform. The onboard sensor was the AlphaUni AS-1300HL long-range multi-platform LiDAR system by CHCNAV. This system uses a 1550 nm Class 1 eye-safe laser, supports 360°full-scan mode, with a maximum range of 1845 m (for targets with >80% reflectivity), a maximum pulse rate of 1.5 MHz, single-shot precision of 15 mm, and repeatability of 10 mm. The system weighs 4.5 kg, consumes 65W of power, and can operate stably in temperatures from −10 °C to +40 °C. It is equipped with an integrated GNSS/IMU (200 Hz) for high-precision positioning and orientation, meeting the requirements for 3D forest point cloud acquisition. Four flights were conducted at an average flight altitude of 80 m and an average speed of 6 m/s, yielding a point cloud density of approximately 350 pts/m^2^. The research workflow included site reconnaissance, ground control point deployment and measurement, flight route planning and LiDAR data acquisition, and indoor data processing and analysis. Based on preliminary data collection and field surveys, the number, H, DBH, and CD of *Catalpa* trees in the plots were measured. Genotypes with fewer than 30 sample trees were excluded, and samples with both LiDAR-derived tree height (LH or *L_H_*) and measured H less than 1.3 m were removed. Ultimately, 78 genotypes comprising a total of 2,899 Catalpa bungei trees were retained for analysis.

DBH, also known as stem diameter, is one of the most fundamental variables in tree measurement. In this study, *Catalpa bungei* was selected as the target species, and the DBH measured at 1.3 m above the ground was used as the standard DBH for individual trees. A total of 2,899 *Catalpa bungei* trees in the study area were measured for DBH. The summary statistics of UAV LiDAR-derived individual tree parameters—LiDAR height (LH or *L_H_*) and LiDAR-derived crown diameter (LCD or *L_CD_*)—are presented in **Table 2**.

**Figure 4:**
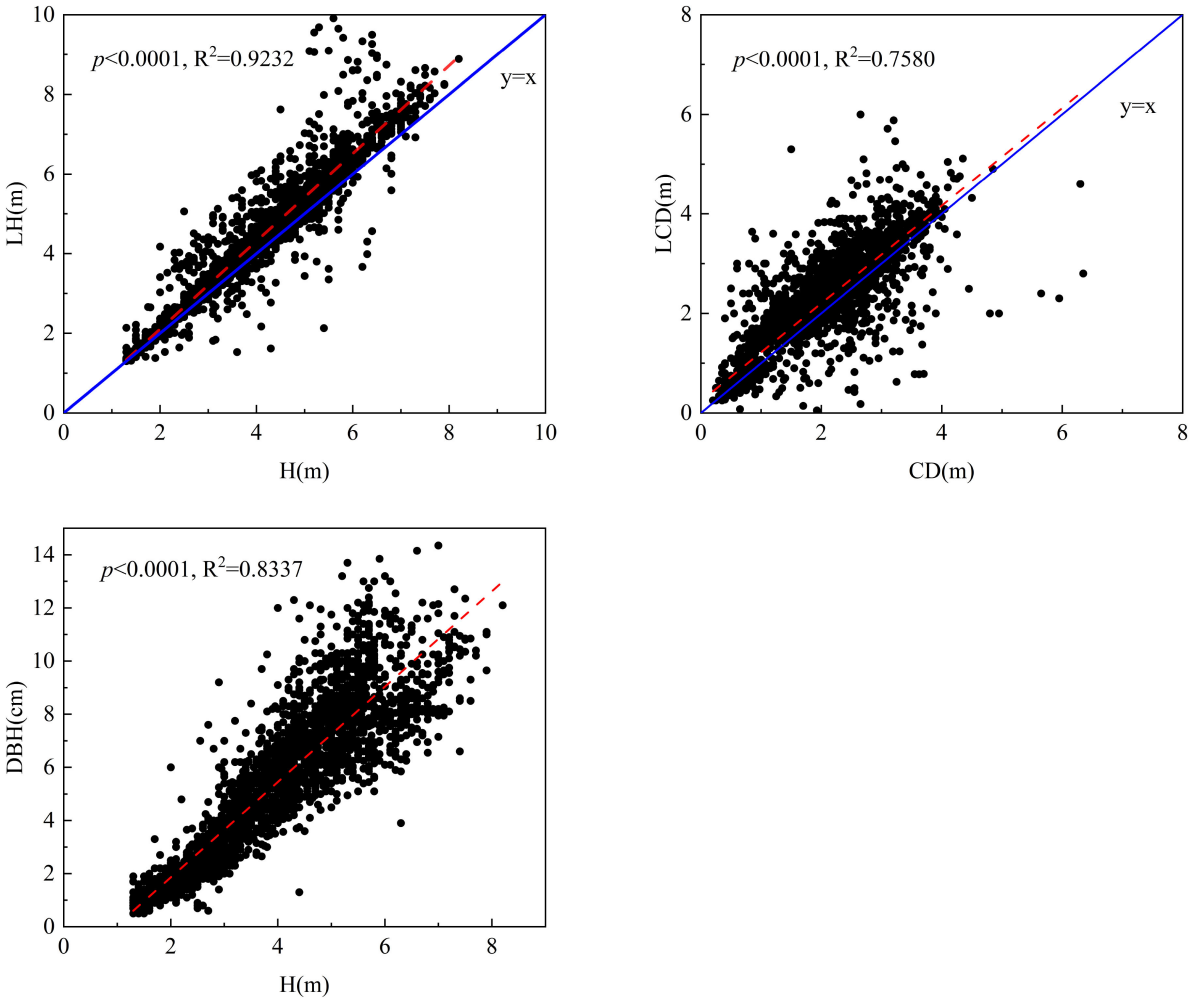
Relationships between LiDAR-derived and field-measured parameters (LH–H, LCD–CD, and DBH–H) for *Catalpa bungei*.

**Figure 4** shows the correlations between LiDAR-derived and field-measured parameters. LH exhibited an excellent linear relationship with H (*R*^2^ = 0.9232, *p<* 0.0001), indicating that UAV LiDAR accurately captured vertical structural information. Similarly, the LCD was strongly correlated with the measured CD (*R*^2^ = 0.7580, *p<* 0.0001), though with slightly higher dispersion due to canopy overlap and segmentation uncertainty. The DBH–H relationship (*R*^2^ = 0.8337, *p<* 0.0001) revealed a robust allometric pattern, confirming that H can serve as a reliable predictor of stem size. Collectively, these results demonstrate that UAV LiDAR-derived structural metrics (LH and LCD) can effectively represent field measurements and thus provide a solid foundation for subsequent NLME-based DBH estimation modeling.

**Figure 5:**
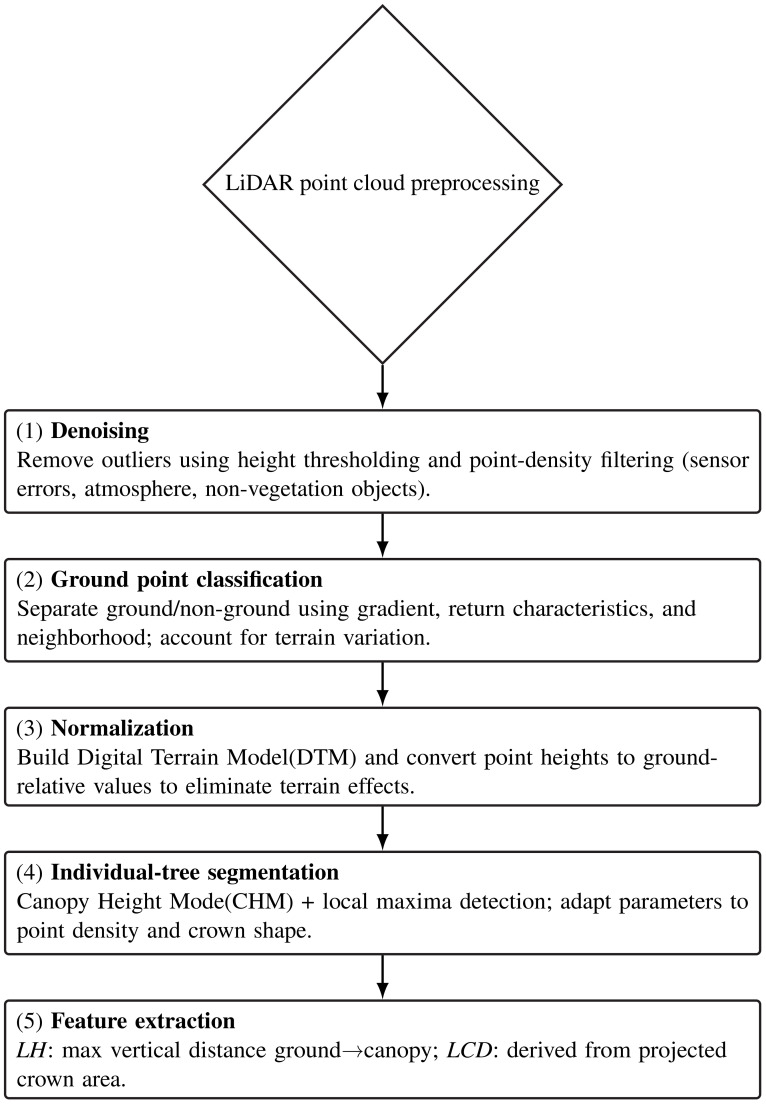
Workflow of LiDAR point-cloud preprocessing and parameter extraction.

**Figure 6:**
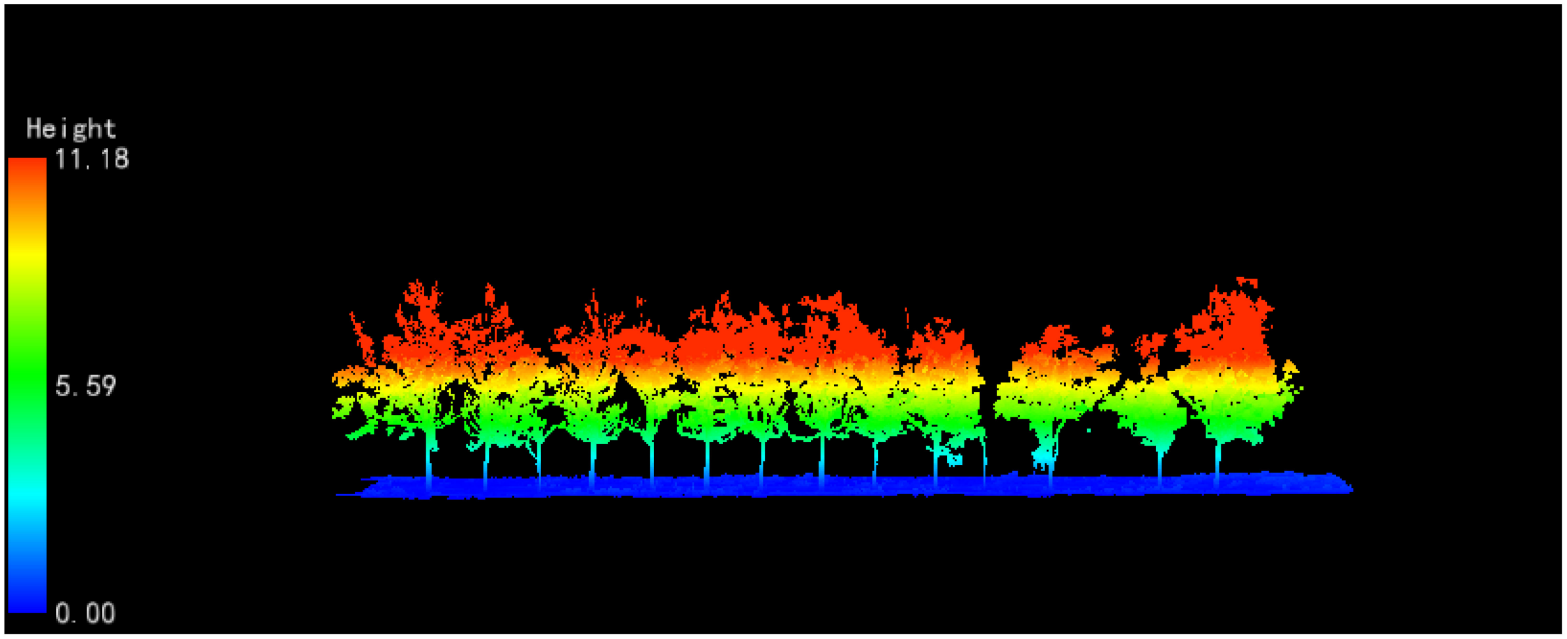
Schematic diagram of airborne LiDAR point cloud data in forested plots.

In this study, Lidar360 software was used to perform systematic preprocessing of airborne LiDAR point cloud data to ensure the accuracy and reliability of extracted LH and LCD parameters. The preprocessing workflow followed the steps below (**Figure 5**):

The schematic diagram of the raw point cloud data is shown below (**Figure 6**).

### 2.3 Predictor variables

In constructing the DBH estimation model based on UAV-LiDAR data,LH and LCD are the most commonly used and stable structural parameters. Studies have shown that LiDAR technology can extract individual tree height with high accuracy (Popescu et al., 2002; Li et al., 2012), and crown width information can also be effectively derived through point cloud-based segmentation methods (Li et al., 2012). Moreover, to avoid model instability and interpretation bias caused by multicollinearity among variables, it is recommended to retain only those variables that are representative and biologically meaningful (Dormann et al., 2013). Therefore, LH and LCD were selected as predictor variables in this study to enhance model stability and generalizability.

### 2.4 Base model selection

To effectively characterize the nonlinear relationship between DBH and LiDAR-derived variables, four commonly used nonlinear models were selected: logistic, exponential, power, and Richards models (Equations 1–4). These models are widely applied in tree growth and biomass prediction studies, as they can reflect different growth patterns from juvenile to mature stages (Zeide, 1993). Among them, the logistic and Richards models exhibit typical S-shaped curves, suitable for simulating rapid early growth, accelerated middle-stage growth, and a stabilizing phase later on (Salas-Eljatib et al., 2021). The exponential model is appropriate for describing monotonically increasing growth trends (Salas-Eljatib et al., 2021; Picard et al., 2025), while the power function captures scale-related relationships between variables (Pilli et al., 2006; Salas-Eljatib et al., 2021). Each model has strengths in biological interpretability, flexibility, and adaptability. As such, they were used as candidate models for comparing performance in DBH prediction, and identifying the optimal fitting function. Through preliminary analysis, models with high coefficients of determination (R²), low root mean square errors (RMSE), and low total relative errors (TRE) were selected as the base models (Equations 5–9). Building upon these, a dummy variable DBH model was developed, followed by the establishment of an NLME DBH model. The formulations of these models are presented as follows:

(1) $$\text{DBH}=\frac{a_{1}}{1+b_{1}exp\;\left(-\left(c_{1}L_{H}+d_{1}L_{CD}\right)\right)}+\epsilon$$

(2) $$\text{DBH}=a_{1}\text{exp }\left(-b_{1}L_{H}-c_{1}L_{CD}\right)+\epsilon$$

(3) $$\text{DBH}=a_{1}L_{H}^{b_{1}}L_{CD}^{c_{1}}+\epsilon$$

(4) $$\text{DBH}=a_{1}\left(1-\text{exp }\left(-b_{1}L_{H}-c_{1}L_{CD}\right)\right)+\epsilon$$

where *DBH* refers to diameter at breast height. Parameters *a*_1_, *b*_1_, *c*_1_, and *d*_1_ are coefficients to be estimated. *L_H_* denotes tree height extracted from LiDAR data, *L_CD_* represents crown width extracted from LiDAR data, and *ϵ* is the residual error.

The best-performing base model was selected based on the following statistical criteria:

(5) $$\bar{e}=\frac{1}{N}\sum e_{i}=\frac{1}{N}\sum_{i=1}^{N}(DBH_{i}-D\hat{B}H_{i})$$

(6) $$\sigma^{2}=\frac{1}{N-1}\sum_{i=1}^{N}{\left(e_{i}-\bar{e}\right)}^{2}$$

(7) $$TRE=100\times \frac{\sum_{i=1}^{N}{\left(DBH_{i}-D\hat{B}H_{i}\right)}^{2}}{\sum_{i=1}^{N}\;DBH_{i}^{2}}$$

(8) $$R^{2}=1-\frac{\sum_{i=1}^{N}{\left(DBH_{i}-D\hat{B}H_{i}\right)}^{2}}{\sum_{i=1}^{N}{\left(DBH_{i}-\bar{DBH}\right)}^{2}}$$

(9) $$\text{RMSE}=\sqrt{{\bar{e}}^{2}+\sigma^{2}}$$

Where: $DBH_{i}$ represents the ith observed DBH; $D\hat{B}H_{i}\;$denotes the ith predicted DBH; $\bar{DBH}$ is the mean of observed DBH values; $e_{i}$ is the prediction error for the ith sample; and N is the total number of samples. The indicators $\bar{e}$, $\sigma^{2}$, TRE, $R^{2}$, and RMSE represent the mean error, error variance, total relative error, coefficient of determination, and root mean square error, respectively. RMSE is defined as a combined measure of the average bias and its variance, and is considered one of the most important metrics for evaluating model performance. These indicators provide a comprehensive assessment of the prediction accuracy and reliability of the model.

### 2.5 Dummy variable model

The dummy variable model is a statistical model widely used for analyzing qualitative or categorical variables. Dummy variables, also known as indicator variables or nominal variables, are primarily used to convert categorical variables into a form suitable for regression analysis, allowing the model to handle non-numeric or unordered categorical data more effectively. In this study, stand density was treated as a qualitative variable, and the dummy variable approach was employed to analyze its influence on the parameters of the DBH growth model.

The dummy variables in this study take values of 0 and 1, assigned as follows:


$$P_{i}=\{\begin{array}{ll} 1, & \text{high}-\text{density planting}\\ 0, & \text{low}-\text{density planting} \end{array},\;i=1$$

Two planting densities—high and low—were considered. The effect of planting density was incorporated into four base model forms using dummy variables, with the corresponding equations expressed as (Equations 10–13).

(10) $$DBH=\frac{a_{i}P_{i}}{1+\left(b_{i}P_{i}\right)exp\;\left(-\left(c_{i}P_{i}L_{H}+d_{i}P_{i}L_{CD}\right)\right)}+\epsilon$$

(11) $$DBH=\left(a_{i}P_{i}\right)\text{exp }\left(-b_{i}P_{i}L_{H}-c_{i}P_{i}L_{CD}\right)+\epsilon$$

(12) $$DBH=\left(a_{i}P_{i}\right)L_{H}^{b_{i}P_{i}}L_{CD}^{c_{i}P_{i}}+\epsilon$$

(13) $$DBH=\left(a_{i}P_{i}\right)\left(1-\text{exp }(-b_{i}P_{i}L_{H}-c_{i}P_{i}L_{CD})\right)+\epsilon$$

*a_i_, b_i_, c_i_, d_i_* (*i* = 1, 2) are parameters related to site conditions in the dummy variable models; *P_i_* denotes the dummy variable, and the other parameters are defined as in the aforementioned base models.

### 2.6 Nonlinear mixed-effects DBH estimation model

The mixed-effects model is formulated based on a regression function that incorporates both fixed-effect and random-effect parameters (Pinheiro et al., 1999). The general form of the single-level mixed-effects model is shown in Equation 14.

(14) $$\left\{ \begin{array}{l} \begin{array}{cc} y_{ij} & =f\left({\text{Φ}}_{i},X_{ij}\right)+\epsilon_{ij},\;i=1,\ldots ,M,\;j=1,\ldots ,n_{i}, \end{array}\\ \begin{array}{cc} {\text{Φ}}_{i} & =A_{i}\beta +\sum_{k=1}^{K}B_{i}^{\left(k\right)}u_{i}^{\left(k\right)}, \end{array}\\ \begin{array}{cc} u_{i}^{\left(k\right)} & ∼N\left(0,\psi^{\left(k\right)}\right),\text{ cov}\left(u_{i}^{\left(k\right)},u_{i}^{\left(l\right)}\right)=0,\;k\neq l,\;k=1,\ldots ,K,\;l=1,\ldots ,K \end{array}\\ \begin{array}{cc} \epsilon_{i} & ∼N\left(0,\;R_{i}\right),\;\epsilon_{i}={\left(\epsilon_{i1},\ldots ,\epsilon_{in_{i}}\right)}^{T} \end{array} \end{array}\right.$$

where $y_{ij}$ is the jth observation of the ith subject with respect to the dependent variable; M is the number of subjects; $n_{i}$ is the number of observations for the ith subject; f(·) is a real-valued and differentiable nonlinear function that depends on a subject-specific parameter vector ${\text{Φ}}_{i}\in {\mathbb{R}}^{p}$ and a covariate vector $X_{ij}\in {\mathbb{R}}^{s}$; $\beta \in {\mathbb{R}}^{p_{0}}$ is the fixed-effect vector; $u_{i}^{\left(k\right)}\in {\mathbb{R}}^{q^{\left(k\right)}}$ is the random-effect vector, assumed to follow a normal distribution with mean zero and variance-covariance matrix $\psi^{\left(k\right)}$. For different k and l, if k≠l, then $u_{i}^{\left(k\right)}$ and $u_{i}^{\left(l\right)}$ are mutually independent. The residual vector $\epsilon_{i}$ is also assumed to follow a normal distribution with mean zero and covariance matrix $R_{i}$, and is independent of the random effects.

When constructing an NLME model with *Catalpa bungei* genotype as the random-effect factor, it is usually necessary to determine the optimal random-effect structure using model selection criteria. The most commonly used criteria are the Akaike Information Criterion (AIC) (Equation 15) and the Bayesian Information Criterion (BIC) (Equation 16). AIC emphasizes model fitting accuracy while accounting for model complexity, making it suitable for relatively small datasets. In contrast, BIC imposes a stricter penalty on the number of model parameters and tends to favor simpler model structures, especially in large-sample settings. Since AIC and BIC differ in focus, combining both metrics provides a more comprehensive assessment of the trade-off between model fit and complexity, enabling more robust model selection. The nonlinear mixed-effects model was implemented using the ‘nlme’ package in R version 4.2.

(15) AIC=2k−ln(L)

(16) BIC=k ln(N)−2 ln(L)

where *k* is the number of model parameters, *N* is the number of samples, and *L* is the value of the likelihood function.

### 2.7 Determination of the variance–covariance Matrix (Ψ) structure among different genotypes

The variance–covariance matrix Ψ for the genotype-level random effects is assumed to be identical for all genotypes and is used to describe the variability in DBH among genotypes. Ψ is assumed to be an unstructured matrix (Calama and Montero, 2005). We assumed the following 2 × 2 variance–covariance matrix:


$$\text{Ψ}=\left(\begin{array}{cc} \sigma_{11}^{2} & \rho_{12}\\ \rho_{21} & \sigma_{22}^{2} \end{array}\right)$$

where *σ_i_*^2^(*i, j* = 1, 2*, i* = *j*) is the variance of the *i*^th^ random effect, and σij(i,j = 1,2,i ≠ j) is the covariance between the *i*^th^ and *j*^th^ random effects.

### 2.8 Determination of the variance–covariance matrix (*R_i_*) structure within the same genotype

We adopted a general framework for modeling the within-group error covariance structure to account for heteroscedasticity and autocorrelation within the same genotype group (Davidian and Giltinan, 2017), both of which are reflected in *R_i_* (Equation 17) (Davidian and Giltinan, 2017; Meng and Huang, 2009):

(17) $$R_{i}=\sigma^{2}G_{i}^{0.5}{\text{Γ}}_{i}G_{i}^{0.5}$$

where *σ*^2^ is a dispersion parameter, also referred to as the scaling factor, which equals the residual variance of the model; *G_i_* is an *n_i_*×*n_i_* diagonal matrix representing heteroscedastic variance within subplot samples; and Γ*_i_* is an *n_i_*×*n_i_* matrix representing autocorrelation within the same genotype. In our data, no autocorrelation pattern was observed among observations; thus, Γ*_i_* was simplified to an *n_i_*× *n_i_* identity matrix.

We evaluated two commonly used variance-stabilizing functions: the exponential function (Equation 18) and the power function (Equation 19), to address variance heterogeneity (Rombouts et al., 2010). The most effective variance function was then selected using the Likelihood Ratio Test (LRT) and the Akaike Information Criterion (AIC) (Pinheiro et al., 1999; Fang and Bailey, 2001):

(18) $$\text{var}\left(\epsilon_{ij}\right)=\sigma^{2}\text{ exp }\left(2\gamma x_{ij}\right)$$

(19) $$\text{var}\left(\epsilon_{ij}\right)=\sigma^{2}x_{ij}^{2\gamma }$$

where *x_ij_* is the selected predictor variable, *L_H_* or *L_CD_*, and *γ* is the parameter to be estimated.

### 2.9 Model estimation

In this study, all variants of the NLME models were fitted using the maximum likelihood estimation method based on the Lindstrom and Bates (LB) algorithm in R software (version 3.4.2). The LB algorithm has been extensively described in previous studies (Pinheiro et al., 1999; Fang and Bailey, 2001; Lindstrom and Bates, 1990). It estimates model parameters through an iterative optimization process that maximizes the likelihood function of the model given the data. The nlme function in R implements this algorithm, supporting the fitting of complex models and simultaneously handling both fixed and random effects. This method is particularly advantageous for analyzing correlated data due to its ability to account for hierarchical or grouped data structures.

### 2.10 Subject-specific prediction

The NLME-based DBH estimation model was used to predict DBH values both with and without random effects. This study focused on prediction models that include random effects, also known as subject-specific or localized models. Localized mixed-effects models are also referred to as calibration models (Meng and Huang, 2009). The calibration process requires prior information—in this study, it refers to the measured DBH values of subsample trees. The prediction of random effects was performed using the empirical best linear unbiased prediction (EBLUP) method (Meng and Huang, 2009; Fang and Bailey, 2001).

(20) $${\hat{u}}_{i}=\text{Ψ}Z_{i}^{T}{\left({\hat{R}}_{i}+Z_{i}\hat{\Psi }Z_{i}^{T}\right)}^{-1}e_{i},=\text{Ψ}Z_{i}^{T}{\left({\hat{R}}_{i}+Z_{i}\hat{\Psi }Z_{i}^{T}\right)}^{-1}\left[y_{i}-f\left(\hat{\beta },u_{i}^{*},x_{i}\right)+Z_{i}u_{i}^{*}\right]$$

where ${\hat{u}}_{i}$ is the q-dimensional predicted random effect vector for the $i^{\text{th}}$ subplot ( i=1,…,M); $u_{i}^{*}$ is the EBLUP vector of the random effect $u_{i}$; f(·) is the NLME model for DBH estimation; $\hat{\beta }$ is theestimated vector of fixed-effect parameters β; $x_{i}$ is the vector of predictor variables; $\hat{\Psi }$ is the estimated variance–covariance matrix of the random effects $u_{i}$ ( i=1,…,M); ${\hat{R}}_{i}$ is the estimated variance–covariancematrix of the residual $e_{i}$; and $Z_{i}$ is an $n_{i}\times q$ design matrix representing the partial derivative of the nonlinear mixed-effects model f(·) with respect to the random effects $u_{i}$. Since the unknown random effects appear on both sides of (Equation 20), ${\hat{u}}_{i}$ cannot be solved by direct algebraic methods. To address this, a three-step iterative algorithm based on EBLUP theory was developed to predict random effects (Meng and Huang, 2009), and a computer program in R was subsequently developed to implement this algorithm (Fu et al., 2017).

### 2.11 Sampling strategies and model calibration evaluation

We used DBH measurements from different numbers of sample trees to predict the random effects in (Equation 20). In general, the more sample trees used in a localized model,the more accurate the prediction (Fu et al., 2017; Xie et al., 2023). Many modeling studies have identified the optimal number of samples required for calibrating NLME models to achieve reliable prediction accuracy.Based on previous studies (Fu et al., 2020), we applied the following four sampling schemes for selecting sample trees from each subplot to capture subject-specific variation in DBH:

1. The 1 to 10 smallest trees per genotype based on DBH.
2. The 1 to 10 largest trees per genotype based on DBH.
3. A random selection of 1 to 10 medium-sized trees per genotype (between the 20th and 80th percentiles of DBH).
4. A random selection of 1 to 10 trees per genotype regardless of size.

We evaluated the prediction performance of each scheme using common statistical metrics, including total relative error (TRE, (Equation 7)), coefficient of determination (R², (Equation 8)), and root mean square error (RMSE, (Equation 9)).

### 2.12 Model evaluation

We employed a leave-one-out cross-validation approach to assess the performance of the NLME models. In each iteration, the *Catalpa* sample of one genotype category was selected as the validation set, while the samples from the remaining 77 genotype categories formed the training set. The DBH model was fitted using the training set to estimate model parameters. These estimated parameters were then used to make predictions on the validation set, and prediction statistics—such as the coefficient of determination (*R*^2^), root mean square error (RMSE), and total relative error (TRE) [see (Equations 5–9)]—were calculated. Once one genotype had been validated, it was returned to the dataset, and another genotype was selected as the new validation set. This process was repeated until each genotype had been used once as the validation set.

## 3 Results

### 3.1 Parametric regression models

#### 3.1.1 Base models

**Figure 7:**
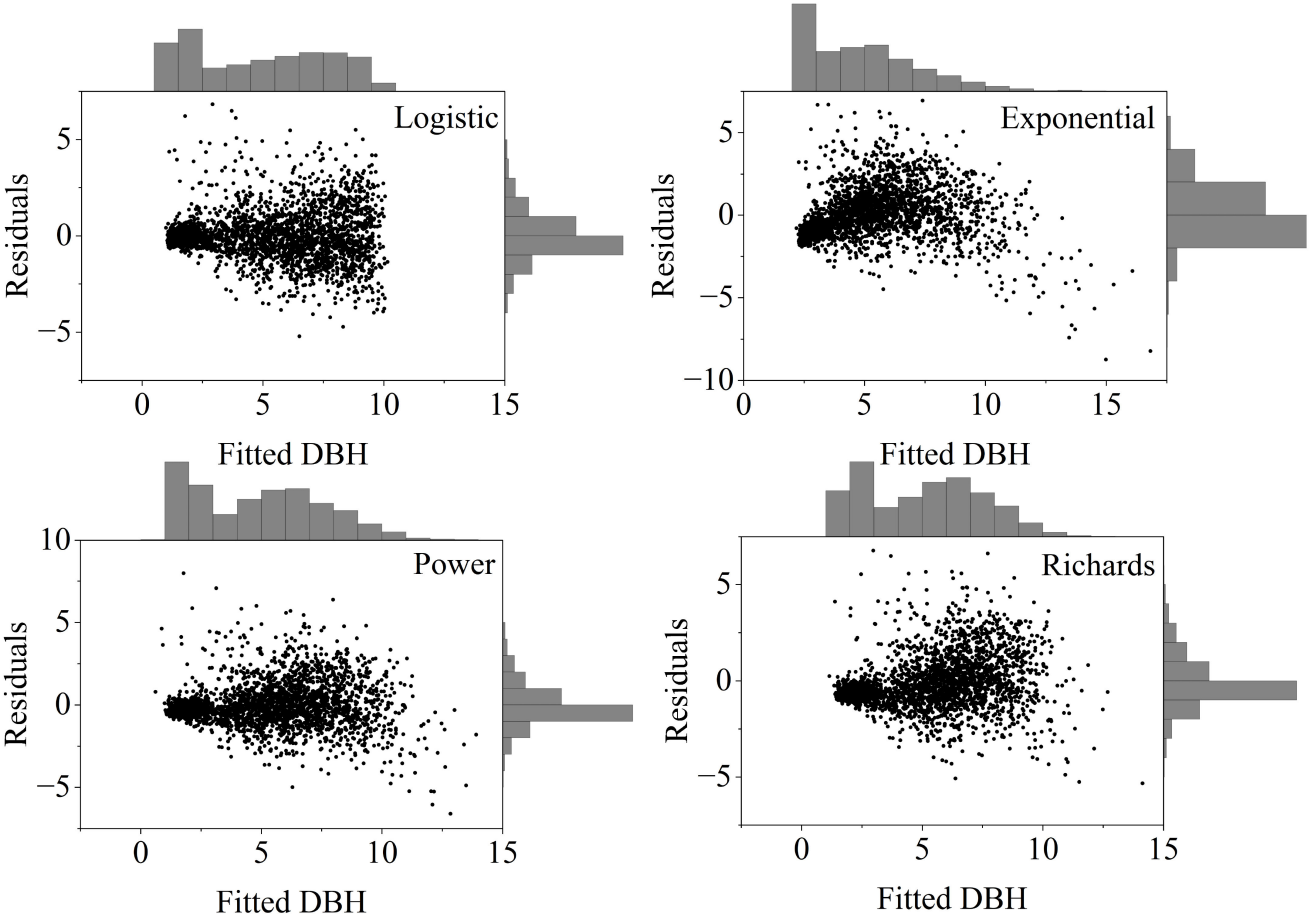
Residual plots of DBH fitted values for the four models and BIC 9150.3254 values were substantially lower, indicating improved predictive accuracy with a more parsimonious model.

During model fitting for DBH, the logistic model (RMSE = 1.2495, *R*^2^ = 0.8326, TRE = 4.9285) and the power function model (RMSE = 1.3120, *R*^2^ = 0.8155, TRE = 5.4605) showed the best accuracy (**Table 3**). Although the logistic model slightly outperformed the power function model, it had a more complex form, and its residual plots showed a radiating pattern (**Figure 7**), indicating more severe heteroscedasticity compared to the power function model. Therefore, the power function model was chosen as the base model for developing the dummy variable DBH prediction models.

**Table 3:** Fitting statistics of the four candidate base models.

Model	Number of parameters	RMSE	R2	TRE	AIC	BIC
Logistic	4	1.2495	0.8326	4.9285	9527.3631	9557.2237
Exponential	3	1.5834	0.7312	8.1569	10898.6786	10922.5671
Power	3	1.3120	0.8155	5.4605	9808.4256	9832.3141
Richards	3	1.3489	0.8049	5.7914	9969.2602	9993.1487

#### 3.1.2 Dummy variable models

To improve the convergence rate of the subsequent NLME models, we simplified the structure of the dummy variable models. The specific forms of the three extended equations are shown below.

(21) $$\text{DBH}=\left(a_{1}+a_{2}\cdot P_{1}\right)L_{H}^{b_{1}}L_{CD}^{c_{1}}+\epsilon$$

(22) $$\text{DBH}=a_{1}L_{H}^{\left(b_{1}+b_{2}\cdot P_{1}\right)}L_{CD}^{c_{1}}+\epsilon$$

(23) $$\text{DBH}=a_{1}L_{H}^{b_{1}}L_{CD}^{\left(c_{1}+c_{2}\cdot P_{1}\right)}+\epsilon$$

Among Equations 21–23, (Equation 22) demonstrated higher accuracy (**Table 4**) and was selected as the base form for the NLME model. The power function dummy variable model significantly improved model performance. Compared to the base power model, the power dummy variable model (Equation 22) reduced RMSE by 11.21%, increased *R*^2^ by 4.78%, and reduced TRE by 22.09%. Additionally, the model’s AIC (9120.4648).

**Table 4:** Fit statistics of three different models (AIC, Akaike’s Information Criterion; BIC, Bayesian Information Criterion).

Model	Number of parameters	RMSE	R2	TRE	AIC	BIC
Model(21)	4	1.1833	0.8499	4.3974	9212.0562	9241.9169
Model(22)	4	1.1648	0.8545	4.2548	9120.4648	9150.3254
Model(23)	4	1.1734	0.8524	4.3204	9163.0142	9192.8748

#### 3.1.3 NLME models

Considering that the model included four parameters (*a*_1_, *b*_1_, *b*_2_, *c*_1_), the corresponding random effects were *u*_1_, *u*_2_, *u*_3_, and *u*_4_. There were 15 possible combinations of random effects in the power dummy variable model. Taking into account the trade-off between model fitting accuracy and the number of parameters, the NLME model with random effects *u*_2_ and *u*_3_ acting on *b*_1_ and *b*_2_ was selected as the optimal nonlinear mixed-effects model.

Ultimately, we selected the NLME model in which random effects were assigned to parameters *b*_1_ and *b*_2_, with AIC = 8980.5630 and BIC = 9028.3400. This model achieved the lowest AIC and BIC values among the candidates while maintaining relatively low model complexity, thereby ensuring successful fitting and validation of the NLME model.

The fitting statistics of the NLME model (*b*_1_ + *b*_2_) were: RMSE = 1.0837, *R*^2^ = 0.8741, TRE = 3.6623. Compared to the power dummy variable model (Equation 22), the NLME model (*b*_1_ +*b*_2_) reduced RMSE by 6.96%, increased *R*^2^ by 2.29%, and reduced TRE by 13.93%. Additionally, AIC = 8980.5630 and BIC = 9028.3400 were also improved.

Most parameter estimates were statistically significant (*p<* 0.05). Even with the introduction of random effects, heteroscedasticity was still present in the DBH estimation model (Equation 24). The final NLME DBH model is as follows:

(24) $$\text{DBH}=a_{1}L_{H}^{\left(b_{1}+\mu_{2}+\left(b_{2}+\mu_{3}\right)\cdot P_{1}\right)}L_{CD}^{c_{1}}+\epsilon$$

where *DBH* denotes the diameter at breast height of *Catalpa* trees; *a*_1_, *b*_1_, *b*_2_, and *c*_1_ are fixed-effect parameters; *L_H_*and *L_CD_*represent LiDAR-derived tree height and crown diameter, respectively; *u*_2_ is the random effect on *b*_1_ due to genotype variation; *u*_3_ is the random effect on *b*_2_ due to genotype variation; *P*_1_ is a dummy variable; and *ϵ* is the residual error.

### 3.2 Parameter estimation

We evaluated two types of variance-stabilizing functions. Among them, the power variance function using *L_H_* as the predictor variable performed best. Although the residual plots did not visually show a clear improvement after introducing the power variance function—possibly due to the large sample size and dense distribution of residual points, which may obscure local structure variations—the AIC value of the model decreased significantly (from 8980.56 to 8384.73), indicating that model fitting improved substantially. Therefore, considering both model evaluation metrics and the rationality of the error structure, the final model retained the power-type variance structure to enhance the robustness of parameter estimation and the overall interpretability of the model. This structure was applied to (Equation 24). After substituting the estimated values of the fixed-effect parameters into (Equation 24), the final NLME DBH estimation model becomes:

(25) $$\text{DBH}=0.84\cdot L_{H}^{\left(1.183+u_{2}+\left(-0.1427+u_{3}\right)\cdot P_{1}\right)}\cdot L_{CD}^{0.2726}+\epsilon$$

where:


$$u_{i}=\left[\begin{array}{c} u_{2}\\ u_{3} \end{array}\right]∼N\left(\left[\begin{array}{c} 0\\ 0 \end{array}\right],{\text{Ψ}}_{1}=\left(\begin{array}{cc} 7.763\text{E}-03 & -4.857\text{E}-03\\ -4.857\text{E}-03 & 5.401\text{E}-03 \end{array}\right)\right),$$

$$\epsilon_{i}={\left(\epsilon_{i1},\ldots ,\epsilon_{in_{i}}\right)}^{T}∼N\;\left(0,\;R_{i}=0.1212\cdot G_{i}^{0.5}{\text{Γ}}_{i}G_{i}^{0.5}\right),$$

$$G_{i}=\text{diag}\left(L_{H_{i1}}^{1.683},\ldots ,L_{H_{in_{i}}}^{1.683}\right),$$

$${\text{Γ}}_{i}=I_{n_{i}},$$

The matrix *I_ni_*is an *n_i_*× *n_i_*identity matrix. All other parameters and predictor variables in this model are defined as previously described.The estimated parameters for (Equation 25) are listed in **Table 5**.

**Table 5:** Parameter estimates and fit statistics of two different models (AIC, Akaike’s information criterion; and LL, log-likelihood).

Parameter type	Parameters	Model(3)	Model(22)	Model(24)	Model(25)
Fixed-effects parameters	*a*1	1.3330	0.9818	0.9502	0.8403
	*b*1	0.7561	1.0795	1.1042	1.1829
	*b*2	–	-0.1434	-0.1463	-0.1427
	*c*1	0.4081	0.2818	0.2759	0.2726
Variance components	*s*1	–	–	1.034E-03	7.763E-03
	*s*2	–	–	2.123E-03	5.401E-03
	*s*12	–	–	-8.155E-04	-4.857E-03
	*γ*	–	–	–	0.8415
	*σ*	–	–	1.1029	0.3482
Model performance	AIC	9808.43	9120.46	8980.56	8384.73
	LL	-4900.21	-4555.23	-4482.28	-4183.37

### 3.3 Subject-specific DBH prediction

**Figure 8:**
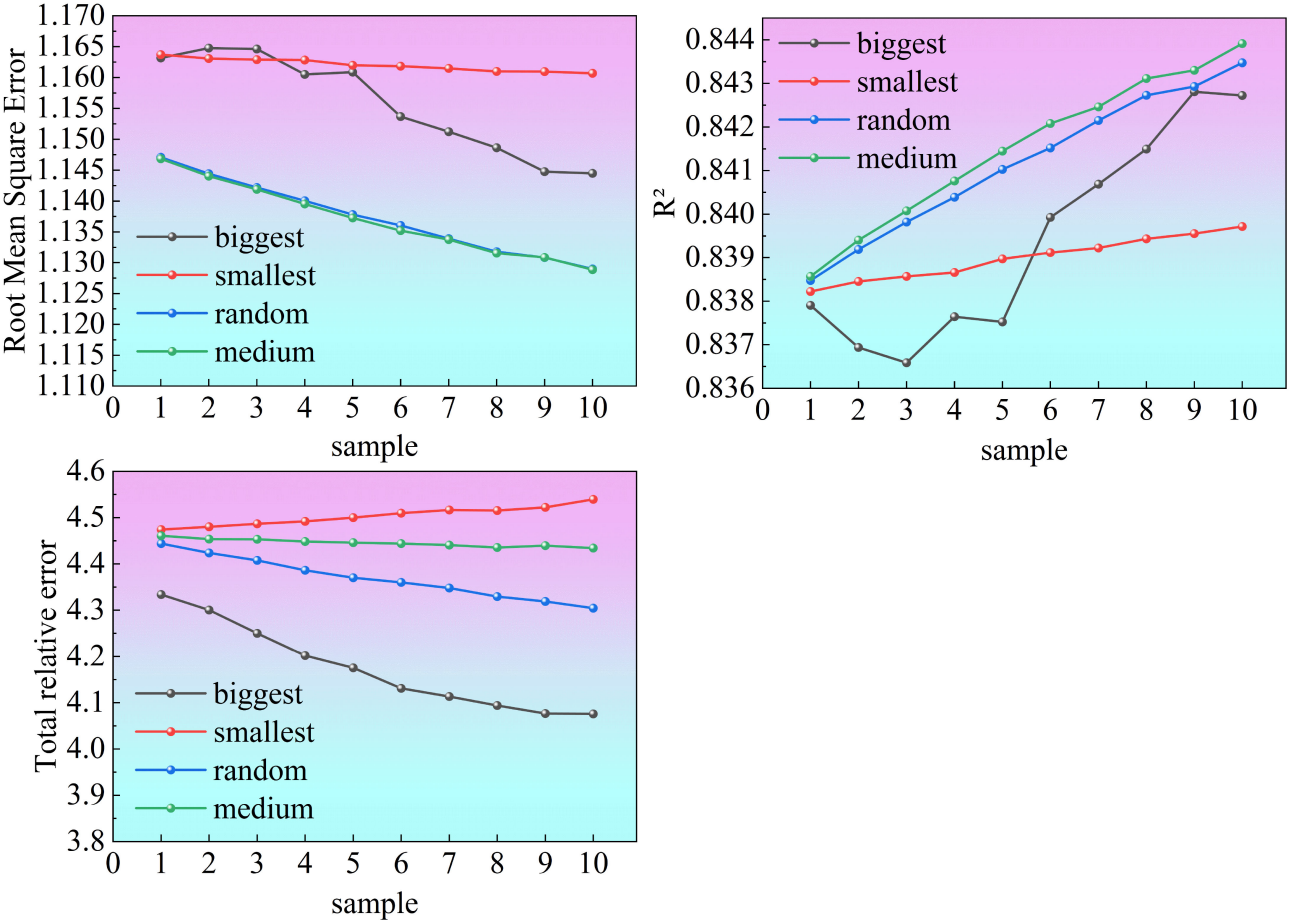
RMSE, R², and TRE of (Equation 25) calibrated using four sampling strategies. Sampling strategies correspond to 1), 2), 3), and 4) in Section 2.11.

The medium and random strategies demonstrated the most stable and consistent performance, as shown in **Figure 8**. Both showed smooth and continuous improvement in all metrics, and their curves were almost identical, suggesting that these two approaches maintained high accuracy and good generalization ability across different sample sizes. Among them, the random strategy achieved comparable accuracy to the medium strategy but offered greater practical advantages due to its simplicity and flexibility in implementation.

It is particularly noteworthy that when the number of randomly sampled trees increased from one to two, the model accuracy showed a noticeable improvement, which was relatively more pronounced compared with the other cases: RMSE decreased to some extent, R² increased, and TRE exhibited a further declining trend. This indicates that under the random sampling strategy, even a very small calibration sample can effectively enhance model performance, while additional samples yield diminishing returns.

In conclusion, the random sampling strategy is recommended, with two sampled trees serving as the optimal calibration configuration. This approach ensures both model stability and prediction accuracy while significantly reducing sampling and computational costs, making it the most practical and scientifically sound choice.

### 3.4 Model evaluation

All statistical models developed in this study—including the base model, dummy variable model, and NLME model—were evaluated using an independent dataset. During evaluation, prediction accuracy was quantified using methods defined by (Equation 5) through (Equation 9), producing key statistical metrics including the coefficient of determination (*R*^2^), root mean square error (RMSE), and total relative error (TRE). These indicators provided important reference points for assessing model performance.

All models were evaluated on independent datasets using three statistical metrics (RMSE, R², and TRE). During model fitting, (Equation 25) achieved *R²* = 0.8624 (5.75% higher than the base power model), *RMSE* = 1.1330 (13.63% lower), and *TRE* = 3.9556 (27.60% lower), indicating that the nonlinear mixed-effects model outperformed traditional parametric models in fitting performance.

Furthermore, to assess model robustness, a leave-one-out cross-validation was conducted. Under the random sampling strategy with two sampled trees, the results were *R²* = 0.8392, *RMSE* = 1.1444, and *TRE* = 4.4238, demonstrating that the calibrated model maintained high predictive accuracy and generalization capability during independent validation.

## 4 Discussion

This study developed a NLME model for predicting the DBH of *Catalpa bungei* using UAV LiDAR data and genotype information.

### 4.1 Methodological advances in DBH modeling

Remote sensing technologies, particularly LiDAR, have substantially advanced forest structural assessment by capturing three-dimensional canopy and tree attributes. Traditional DBH models rely on H and CD as key predictors, but often assume population homogeneity. While effective in some contexts, such models overlook genetic variability and management-induced differences that influence tree growth.

The present study introduced genotype as a random effect into the NLME framework, enabling the model to capture intraspecific variation that conventional approaches ignore. Compared with previous LiDAR-based DBH models that incorporated only plot-level or topographic effects (Fu et al., 2020; Yang et al., 2020), our model provides a more refined characterization of growth variability. This innovation allowed us to better explain differences among individuals growing under identical site conditions but with distinct genetic backgrounds. The resulting improvement in predictive performance demonstrates the value of embedding genetic heterogeneity in forest structural modeling.

Beyond genotype, the model structure integrated LiDAR-derived variables (H and CD) with a planting density dummy variable to reflect management effects. This combination enhanced explanatory power and improved generalization. While prior studies have emphasized structural predictors or error-in-variable frameworks, they often remained within fixed-effects models (Fu et al., 2018). By contrast, the NLME approach adopted here not only accommodates structural and management variables but also provides a robust statistical basis for incorporating biological diversity. This methodological flexibility suggests broad applicability across species and ecosystems where genetic and management factors shape tree allometry.

### 4.2 Challenges and perspectives in LiDAR–field data alignment

Although the NLME model demonstrated superior performance under calibration conditions, this advantage largely depends on the high-precision alignment between LiDAR data and field measurements. In practical applications, accurately matching LiDAR-derived tree structural parameters with field-measured DBH remains challenging, especially in degraded stands or areas with complex and overlapping canopy structures. Crown occlusion, sparse point density, and incomplete returns often lead to mismatches between individual trees, thereby affecting model calibration accuracy.

To minimize such errors, future studies should aim to maximize the precision of LiDAR data processing during acquisition and matching, and integrate high-accuracy GNSS positioning systems to assist in spatial registration of individual trees. In addition, several strategies can be considered:

- using high-density UAV LiDAR data combined with accurately measured ground control points for geometric correction;
- recording tree coordinates or stem positions simultaneously during field measurements;
- applying robust tree-matching algorithms based on multi-attribute similarity (e.g., H, CD, spatial proximity);
- adopting probabilistic or fuzzy matching methods to address uncertain correspondences.

These measures can help ensure the spatial consistency and reliability of calibration samples, thereby further improving the predictive accuracy and practical applicability of NLME-based models.

### 4.3 Comparison with existing studies

Previous studies have shown that NLME models can effectively capture forest structural or spatial heterogeneity by incorporating plots or sites as random effects (Fu et al., 2020; Zhang et al., 2021; Zhou et al., 2023). However, these approaches generally assume population homogeneity and fail to adequately account for the influence of intra-specific genetic variation on tree allometric relationships. In contrast, this study introduces genotype as a random effect within the NLME framework, extending mixed-effects modeling from the environmental to the genetic level. This innovation not only significantly improved model accuracy (*R*^2^ = 0.8624, RMSE = 1.1330, TRE = 3.9556) but also enhanced the biological interpretability and ecological applicability of the model by linking LiDAR-derived structural parameters with genotypic growth variation.

Moreover, unlike the calibration strategies adopted by (Fu et al., 2020; Zhou et al., 2023), which relied on selecting the largest or smallest individuals, our findings demonstrate that random sampling can achieve stable and highly accurate predictions with minimal field effort. This result stems from the hierarchical structure of the NLME model and the pronounced genetic heterogeneity within the stand. Incorporating genotype as a random effect enables the model to absorb systematic variation among individuals, making randomly selected samples more representative of overall growth variability. In contrast, extreme sampling (e.g., selecting only the largest or smallest trees) may bias calibration toward specific genotypes or microsite conditions, thereby reducing model generalization. Overall, random sampling achieves an optimal balance between representativeness, accuracy, and field efficiency, underscoring the advantages of genotypebased random-effect modeling for accurate and scalable DBH estimation in genetically diverse forest stands.

### 4.4 Ecological implications of accurate DBH estimation

DBH is widely recognized as the most reliable measurable tree attribute for estimating aboveground biomass (AGB). Errors in DBH estimation propagate into substantial uncertainties in biomass and carbon stock assessments at stand or regional scales. Small deviations can lead to significant over- or underestimation of carbon sequestration potential, undermining both scientific assessments and climate policy mechanisms such as carbon accounting and trading. By improving DBH prediction accuracy, our genotype-informed NLME model contributes directly to more reliable carbon stock estimation and climate change mitigation strategies.

In addition to biomass, DBH is often used as a proxy for biodiversity- and habitat-related indicators. Larger trees typically support greater structural complexity and offer habitat niches for numerous organisms. Accurate estimation of DBH distributions therefore improves our ability to evaluate forest structural diversity, habitat quality, and ecosystem services. By reducing prediction uncertainties, the model strengthens the reliability of ecological indicators that reflect forest health and biodiversity.

The explicit incorporation of genotype is particularly relevant for ecological monitoring. Forest indicators derived from remote sensing often reflect spatial variability, but genetic variability is rarely considered. Our findings show that genetic diversity exerts measurable effects on tree structure, suggesting that ignoring it may underestimate variability in forest productivity and resilience. Genotype-sensitive modeling thus provides a pathway toward integrating biological diversity into remote sensing indicators, a step that can enrich ecological monitoring frameworks and improve our understanding of forest adaptive capacity under environmental change.

### 4.5 Practical considerations for monitoring and sampling

A key component of this study was the evaluation of calibration strategies for mixed-effects models. Four sampling approaches were tested, and random sampling consistently emerged as the most efficient, maintaining accuracy while minimizing fieldwork. Notably, even minimal random sampling (two trees) significantly improved model performance, highlighting the practicality of this approach for large-scale surveys.

This insight has strong operational implications. UAV LiDAR surveys increasingly cover large and remote areas where extensive ground measurements are impractical. By adopting efficient calibration strategies, forest monitoring programs can optimize resource allocation while ensuring data quality. The proposed methodology thus offers a practical template for combining remote sensing with limited ground calibration, applicable to genetically diverse plantations as well as heterogeneous natural forests.

### 4.6 Transferability, limitations, and future directions

Although this study focused on *Catalpa bungei*, the proposed methodological framework is broadly transferable. The flexibility of the NLME model allows incorporation of different random effects, such as species identity, site conditions, or management regimes, making it adaptable across diverse forest ecosystems.

While the proposed genotype-informed NLME framework performed exceptionally well in *Catalpa bungei* plantations, its broader applicability across different tree species and forest types warrants further investigation. Tree species with contrasting growth architectures, diverse reproductive strategies, or more complex interspecific competition may exhibit distinct genotype–phenotype interactions that alter allometric scaling patterns. Extending this framework to natural or seed-propagated forests could therefore reveal new insights into how genetic diversity interacts with environmental heterogeneity to shape individual tree growth. Future studies should explore these dynamics by incorporating additional physiological, climatic, and soil factors, thereby testing the robustness, adaptability, and ecological generality of the proposed modeling approach.

Nevertheless, we acknowledge several limitations. First, while genotype was explicitly included, further validation across multiple species and regions is needed to assess generalizability. Second, only two structural variables (H and CD) were derived from UAV LiDAR; incorporating additional metrics such as crown volume, canopy density, or stand-level heterogeneity may further improve performance. Third, environmental covariates such as soil type, water availability, and microclimate were not included but could be integrated in future studies to capture broader ecological variability. Finally, uncertainties in this study may arise from both observational and sampling sources. Field-measured DBH and LiDARderived structural variables are inevitably affected by positional mismatches, sensor noise, and scaling inconsistencies, which can propagate into model predictions (Richardson et al., 2008). Moreover, variations in sampling intensity and the representativeness of field plots may introduce additional uncertainty, particularly in genetically diverse or structurally heterogeneous stands. Similar to the challenges faced in long-term meteorological monitoring, where gap-filling and interpolation can lead to systematic biases (Shen et al., 2017), minor inconsistencies between field and LiDAR observations may distort calibration accuracy. Although rigorous data preprocessing, filtering, and model fitting were implemented to minimize these effects, the influence of measurement and sampling uncertainty cannot be entirely eliminated. Future work should therefore emphasize enhanced field–LiDAR co-registration accuracy and uncertainty quantification to further improve model robustness and transferability.

Future research should thus focus on expanding the model’s scope across species, ecosystems, and geographic regions. Integrating more detailed LiDAR-derived indicators and environmental data would not only enhance model accuracy but also allow exploration of genotype–environment interactions. Moreover, linking DBH predictions with higher-level ecological processes (e.g., carbon dynamics, resilience under climate extremes) could provide even more powerful insights for ecological monitoring and indicator development.

### 4.7 Contributions to ecological indicators and monitoring

By bridging UAV LiDAR data, advanced statistical modeling, and genetic information, this study contributes to the development of ecological indicators in several ways. First, it strengthens the accuracy of DBH estimation, which underlies key indicators of biomass, carbon stocks, and forest productivity. Second, it demonstrates that incorporating genetic diversity improves the ecological relevance of structural indicators, aligning them more closely with biodiversity and resilience assessments. Third, it provides practical guidance for sampling and calibration in UAV-based monitoring, thereby enhancing the efficiency and scalability of ecological surveys.

Together, these contributions advance the role of remote sensing in ecological monitoring, bridging the gap between individual-level measurements and ecosystem-level assessments.

## 5 Conclusion

This study developed a NLME model for predicting the DBH of *Catalpa bungei* by integrating UAV LiDARderived structural variables with genotype information. DBH serves as a key indicator of tree growth, biomass, and stand structure, and its accurate estimation is essential for evaluating forest productivity, ecosystem health, and carbon storage.

Incorporating genotype as a random effect markedly improved model accuracy and reduced uncertainty, emphasizing the importance of genetic diversity in shaping tree allometry and growth variability. LH and LCD effectively represented structural attributes related to DBH, enabling robust predictions across different stand conditions. Random sampling proved to be the most efficient calibration strategy, achieving high accuracy with minimal fieldwork.

Overall, the genotype-sensitive NLME framework provides a scalable and ecologically meaningful approach to improving DBH estimation, supporting precise forest inventory, carbon accounting, and sustainable management of genetically diverse forest ecosystems.

## Data Availability

The raw data supporting the conclusions of this article will be made available by the authors, without undue reservation.
